# Three-Compartment Pharmacokinetics of Inhaled and Injected Sinapine Thiocyanate Manifest Prolonged Retention and Its Therapeutics in Acute Lung Injury

**DOI:** 10.3390/pharmaceutics17070909

**Published:** 2025-07-14

**Authors:** Zixin Li, Caifen Wang, Huipeng Xu, Qian Wu, Ningning Peng, Lu Zhang, Hui Wang, Li Wu, Zegeng Li, Qinjun Yang, Jiwen Zhang

**Affiliations:** 1 College of Pharmacy, Anhui University of Chinese Medicine, Hefei 230012, China; zixinli201475@163.com; 2 Center for Drug Delivery Systems, Shanghai Institute of Materia Medica, Chinese Academy of Sciences, Shanghai 201210, China; wangcaifen@simm.ac.cn (C.W.); hpxu713@163.com (H.X.); 19834519869@163.com (Q.W.); pnn19863823375@163.com (N.P.); wuli@simm.ac.cn (L.W.); 3 Jiangsu Yungou Pharmaceutical Technology Co., Ltd., Yangtze Delta Drug Advanced Research Institute, Nantong 226133, China; 4 College of Pharmacy, Shenyang Pharmaceutical University, Shenyang 110016, China; 5 College of Chinese Medicine, Anhui University of Chinese Medicine, Hefei 230038, China; 18715028922@163.com (L.Z.); 15077916827@163.com (H.W.); 6 Department of Respiratory, The First Affiliated Hospital of Anhui University of Chinese Medicine, Hefei 230031, China; li6609@126.com

**Keywords:** sinapine thiocyanate, dry powder inhaler, pharmacokinetic, acute lung injury, MAPK signaling pathway

## Abstract

**Background**: Acute lung injury (ALI) is driven by inflammatory cascades and reactive oxygen species (ROS) generation, with the progression to severe cases markedly increasing mortality. Sinapine thiocyanate (ST), a bioactive natural compound isolated from Sinapis Semen Albae (SSA), demonstrates both anti-inflammatory and antioxidant pharmacological activities. However, no monotherapeutic formulation of ST has been developed to date. A dry powder inhaler (DPI) enables targeted pulmonary drug delivery with excellent stability profiles and high inhalation efficiency. **Methods**: ST was purified and prepared as inhalable dry powder particles via an antisolvent crystallization technique. The therapeutic mechanisms of ST against ALI were elucidated by network pharmacology and pharmacokinetic analyses, with the therapeutic efficacy of the ST DPI in ALI mitigation being validated using LPS-induced rat models. **Results**: The ST DPI showed ideal aerodynamic characteristics. Notably, ST exhibited a three-compartment (triexponential) pharmacokinetic profile following both intravenous tail vein injection and inhalation administration. Furthermore, the inhaled formulation displayed a prolonged systemic residence time, which confers therapeutic advantages for pulmonary disease management. Furthermore, the inhalation administration of ST demonstrated a 2.7-fold increase in AUC compared with oral gavage, with a corresponding enhancement in systemic exposure. The ST DPI formulation demonstrated significant therapeutic efficacy against ALI in rats by downregulating inflammatory cytokines and modulating oxidative stress levels, mechanistically achieved through the MAPK-mediated regulation of cellular apoptosis via a positive feedback loop. **Conclusions**: The unique triexponential plasma level profiles of an ST DPI provide a promising pharmacokinetics-based therapeutic strategy for ALI, leveraging its marked efficacy in attenuating inflammation, oxidative stress, and pulmonary injury.

## 1. Introduction

Sinapis Semen Albae (SSA) refers to the desiccated and fully developed seeds of *Sinapis alba* L., which was initially documented by Ming Yi Bie Lu (Supplementary Records of Famous Physicians) [1,2]. Sinapine thiocyanate (ST) is the marker compound of SSA and is also recognized as the active pharmacological component for its antitussive, antiasthmatic, and expectorant effects. Modern pharmacological studies show that ST improves vascular endothelial dysfunction in hypertensive patients by inhibiting NLRP3 inflammasome activation [3,4] and treats bronchial asthma by reducing PTGS2, MMP-9, and IL-2 levels [5]. ST exhibits potent antioxidant activity and mitigates Aβ protein-induced toxicity with therapeutic potential for Alzheimer’s disease [6,7]. The absolute bioavailability of ST is only 1.84% with oral administration [8] and has not yet been developed into a single-component preparation for pharmaceutical applications.

Acute lung injury (ALI) is a diffuse pulmonary inflammatory response caused by harmful substance inhalation, infections, and trauma, leading to alveolar endothelial barrier damage [9,10]. It is characterized by acute respiratory failure, tachypnea, refractory hypoxemia, reduced lung compliance, and bilateral alveolar infiltrates on imaging [11]. The pathogenesis of ALI involves inflammation and the production of ROS [12,13]. Glucocorticoids are commonly used in clinical practice to treat ALI [14,15,16]. However, higher doses and the long-term use of glucocorticoids can lead to infections due to immune dysregulation and adverse effects on multiple organs and systems [17,18,19]. On the other hand, studies have demonstrated that numerous natural compounds exhibit potential in preventing ALI, with certain natural ingredients already approved by the National Medical Products Administration of China. Consequently, the development of safer and more effective therapeutic strategies for ALI remains a pivotal research priority, and natural constituents offer a highly promising avenue.

Due to unique physiological barriers, oral or intravenous drugs often achieve low pulmonary distribution, limiting efficacy and risking systemic adverse effects with dose escalation. Pulmonary drug delivery is a non-invasive method and directly targets the lungs via the respiratory tract as the route of administration. Leveraging the dense capillary network surrounding the alveoli, drugs are rapidly absorbed into circulation, bypassing the first-pass effect and improving bioavailability [20]. Pulmonary administration effectively treats various lung diseases, including asthma, COPD, and ALI [21]. A dry powder inhaler (DPI) delivers micronized active pharmaceutical ingredients to the deep lung to achieve a high inhalation efficiency, excellent stability, ease of use, and lack of pollution [22].

In this study, ST was prepared as a new formulation to attenuate ALI. The interaction mechanism of ST via network pharmacology revealed its pharmacological action through the suppression of the MAPK pathway-mediated pro-apoptotic positive feedback loop. Then, ST was engineered as an inhalable dry powder formulation demonstrating favorable aerodynamic properties and physicochemical stability. The pulmonary delivery of ST powder significantly enhanced bioavailability compared with oral administration and exhibited a unique three-compartment pharmacokinetic profile. The ST DPI demonstrated promising efficacy in the treatment of ALI.

## 2. Materials and Methods

### 2.1. Materials

ST was extracted and purified to a purity of 97.02% (Supplementary Materials). Ethanol and methanol were procured from Sinopharm Chemical Reagent Co., Ltd. (Shanghai, China). Ethyl acetate, HPLC-grade methanol, and acetonitrile were supplied from Meryer Chemical Technology Co., Ltd. (Shanghai, China). Phosphate-buffered saline (PBS) and Roswell Park Memorial Institute 1640 (RPMI 1640) were provided by Dalian Meilun Biotechnology Co., Ltd. (Dalian, China). Human lung cancer cells (A549) were obtained from the Shanghai Cell Bank of the Chinese Academy of Sciences (Shanghai, China) and mouse alveolar macrophages (MH-S) were provided by ATCC (Manassas, VA, USA). Lipopolysaccharides (LPS) from E. coli (O55:B5) was obtained from Merck Ltd. (Darmstadt, Germany). Isoflurane was purchased from Hebei Jindafu Pharmaceutical Co., Ltd. (Hebei, China). Zoletil 50 was purchased from BAILAIYUAN Biotechnology Co., Ltd. (Tianjin, China). Rat interleukin-1β (IL-1β), interleukin-6 (IL-6), and tumor necrosis factor-α (TNF-α) ELISA Kit were obtained from Wuhan Jiyinmei Biotech Co., Ltd. (Wuhan, China). SOD and MDA Assay Kit were purchased from Nanjing Jiancheng Bioengineering Research Institute (Nanjing, China). Nuclear factor kappa-B (NF-κB), nuclear factor erythroid 2-related factor 2 (Nrf2), caspase-3, caspase-9, and c-Jun N-terminal kinase 1 (JNK1) were supplied by Abcam (Cambridge, UK). MAPK kinase kinase 1 (MEKK1) and p38 mitogen-activated protein kinase (p38) was supplied by Bioss (Beijing, China), and glyceraldehyde-3-phosphate dehydrogenase (GAPDH) was supplied by Zsbio (Beijing, China).

### 2.2. Animals

The SD rats were provided by Jiangsu Huachuang Sino Pharmaceutical Technology·Co., Ltd. (Taizhou, China). The experimental protocol received ethical approval from the Institutional Animal Care and Use Committee (IACUC) at Shanghai Institute of Materia Medica (2024-03-ZJW-52). All animal experiments and husbandry practices were conducted strictly within the established IACUC guidelines and regulations.

### 2.3. Network Pharmacology

ST has not been investigated yet for its therapeutic applications in ALI and no single-component preparation has been developed. Given the demonstrated anti-inflammatory and antioxidant properties of ST, a network pharmacology approach was first employed to analyze the feasibility and potential mechanisms of ST in the management of ALI.

#### 2.3.1. Prediction and Screening of Potential Targets for ST in ALI

First, the MOL file of ST was obtained from the TCMSP website [23]. This file was then converted to SMILES format using an online tool (https://www.novopro.cn/tools/mol2smiles.html, accessed on 9 May 2024), and the resulting SMILES string was used to search for predicted targets in the Swiss Target Prediction database [24]. ALI-related targets were systematically curated from the GeneCards database (filtered with a relevance score threshold > 15) and the OMIM database, while no ALI-associated targets were retrieved from DISGENET. An intersection diagram was then created to visualize the genes related to ST and ALI.

The intersection target of ST and ALI was introduced at the STRING online level to construct a protein–protein interaction (PPI) network. TSV files of intersection targets obtained from STRING were imported into the Cytoscape 3.10.2 software, a protein PPI visualization network was established, and the network topology was analyzed by a network analysis plug-in. Targets with between centrality, closeness centrality, and degree values more incredible than the median were selected as potential core targets for treating ALI with ST. The larger the target circle, the darker its color, and the more crucial the associated explanation.

#### 2.3.2. Kyoto Encyclopedia of Genes and Genomes (KEGG) and Gene Ontology (GO) Enrichment Analyses

The intersecting genes were uploaded to the DAVID database, with “Homo sapiens” selected, then analyzed on GO in terms of biological process (BP), cellular component (CC), molecular function (MF), and KEGG pathways to obtain the results. The BioInformatics platform (http://www.bioinformatics.com.cn/, accessed on 13 May 2024) created GO bar plots and KEGG enrichment bubble charts, which showed the molecular functions, involved biological processes, and related cellular components of core targets.

#### 2.3.3. Molecular Docking

By constructing a component–target pathway network diagram of ST and ALI disease targets and pathways, the PPI and related literature can be combined to screen targets. The 2D structure of the target component ST was retrieved from the PubChem database, and the ligand was converted to a PDBQT file. The gene targets were searched in the PDB database (https://www.rcsb.org/, accessed on 19 May 2024), and PyMOL 2.5.4 removed water and impurities and saved them as a PDB file. The ligand ST conformation was energy minimized using Chem3D 23.0.1, which ensured the ligand adopted a more stable conformation prior to docking. The receptors were hydrogenated with AutoDockTools 1.5.7 and saved as PDBQT files. The files were formatted by OpenBabel 3.1.1, and molecular docking was performed by MultiTomulti, with binding energy calculated and recorded.

### 2.4. Preparation and Characterization of ST DPI

#### 2.4.1. Ultrasonic-Assisted Antisolvent Preparation of ST DPI

The ST was extracted and purified (Supplementary Materials). Then, it was structurally characterized by ^1^H NMR and identified by HPLC analyses in comparison to a reference material. ST DPI was prepared via an ultrasound-assisted anti-solvent method utilizing methanol/ethyl acetate (1:10 *v*/*v*) with supersaturation-driven crystallization. Process parameters (10 min reacting, 1500 rpm stirring, and 10 min sonicating) were optimized by selecting an appropriately sized rotor supplemented with ultrasonic to control needle-shaped crystal morphology through axial fragmentation and preferential crystal plane growth. The obtained particles were vacuum-filtered and dried at 60 °C for 4 h to obtain ST DPI.

#### 2.4.2. Characterization of ST DPI

ST DPI and API were characterized to confirm whether the properties of the ST DPI were altered. The morphological characteristics of ST DPI and API were examined by scanning electron microscopy (SEM, Phenom Pharos G2; Thermo Fisher Scientific, Waltham, MA, USA). All samples were attached to the sample rack with aluminum-based double-sided adhesive tape, and all images were taken on an SED detector mode with a beam intensity of 5 kV. The particle size distributions of ST DPI and API were measured by laser diffraction (PAQXOS-HELOS|RODOS, Sympatec GmbH, Clausthal-Zellerfeld, Germany) with an R1 lens (0.1–35 μm), with a sampling speed as 70 mm/s, the pressure at 4.5 bar, and the vacuum at 20 mbar. Dynamic moisture adsorption (DVS) was used to determine the hygroscopicity of the powder and the temperature was adjusted to 25 °C. The humidity increased from 0 to 95% with a 5% gradient, then decreased from 95% to 0 with the same gradient to obtain the diagram of relative mass change vs. relative humidity change. A thermal gravimetric analyzer (TGA, PerkinElmer TGA 8000, Shelton, CT, USA) was used to study the thermal stability of the powder with the temperature range from 30 to 200 °C, the scanning rate at 10 °C/min, and the nitrogen flow rate at 20 mL/min. Differential scanning calorimetry (DSC, PerkinElmer DSC 8500, Shelton, CT, USA) was employed to analyze the stability of the samples. The samples were programmed from 25 to 300 °C at a rate of 10 °C/min at a nitrogen flow rate of 30 mL/min, and the heat absorption and release curves of the samples were drawn. D8 advanced X-ray diffractometer (XRD, Brooke (Beijing Technology, Beijing, China) was used to study the crystal structure of the materials. Fourier transform infrared spectroscopy (FTIR, Nicolet iS50, Thermo Fisher Scientific, USA) was employed to analyze the functional group composition of the samples. The spectra of the samples were recorded in the wavenumber range of 4000–400 cm^−1^ to determine the presence and characteristics of various functional groups.

#### 2.4.3. In Vitro Deposition Study

In vitro deposition of ST DPI was determined via the next generation impactor (NGI, NRH-ZJQ-160, Beijing HuiRongHe Technology, Beijing, China), with a flow rate of 60.23 L/min and running time at 4.0 s. To calculate the percentage of the particle dose under 5 μm relative to the ejected dose, the ST deposited in the inhalation device, adapter, throat, pre-separator, and the 8 collecting trays were collected with methanol and analyzed by HPLC (Supplementary Materials). The key parameters like fine particle dose (FPD), emitted dose (ED), fine particle fraction (FPF), median aerodynamic diameter (MMAD), and geometric standard deviation (GSD) values were calculated using the inhalation formulation evaluation data analysis software 1.1.2.27 (Beijing HuiRongHe Technology, Beijing, China).

The emptying rate was calculated by first accurately weighing the empty capsules and recording their mass as *W*_1_, followed by filling the capsules with ST DPI powder and recording the total mass as *W*_2_, and the mass of the empty capsules along with the residual drug was measured as *W*_3_ after the NGI testing. Delivered dose uniformity (DDU) refers to the degree of deviation between multiple measured delivered doses and their average, ensuring that the drug dose delivered to the lungs remains consistent with each inhalation.

Emptying rate = (*W*_2_ − *W*_3_)/(*W*_2_ − *W*_1_) × 100% (1)

### 2.5. Pharmacokinetic Study

The bioavailability of ST was evaluated in eighteen Sprague–Dawley (SD) rats (250 ± 20 g), which were randomly divided into three groups (*n* = 6). The animals received ST at a dose of 16 mg/kg via three administration routes: oral gavage (ST solution), pulmonary inhalation (ST DPI), and intravenous injection (ST solution). The rats were fasted for 12 h prior to and 4 h following drug administration, with free access to water throughout the study. Blood samples (0.25 mL) were collected from the orbital vein at 5, 10, 15, 30, and 45 min and 1, 2, 4, 6, and 10 h post administration. The samples were placed in heparin-coated centrifuge tubes (1% heparin sodium salt, 40 μL) and centrifuged at 3500 rpm for 10 min. The supernatant was collected, extracted, and stored at −20 °C for subsequent HPLC-MS/MS analysis.

The content of ST in plasma was determined by a validated HPLC-MS/MS (Agilent 6460) method on an Agilent ZORBAX Eclipse Plus C_18_ (4.6 mm × 100 mm, 3.5 μm) at 45 °C. The mobile phase was regulated to 0.1% formic acid water (A) and 0.1% acetonitrile (B) at a flow rate of 0.6 mL/min, with the gradient elution as follows: 0–1 min at 10% B, 1–2 min at 10–60% B, 2–4 min at 60–80% B, 4–4.5 min at 80% B, 4.5–6 min at 80–10% B, 6–8 min at 10% B, and 5 μL for analysis. Mass spectrometry was conducted using an electrospray ion source of positive and negative ions (+ESI of ST and -ESI of CAP) and a multiple reaction monitoring (MRM) mode. The ion-selective channels selected were ST (*m*/*z* 310.1/251.0) and chloramphenicol (CAP, *m*/*z* 320.9/151.9, internal standard), the voltage and collision energy of ST were set as 150 V and 16 eV; meanwhile, CAP was set as 50 V and 20 eV. All validation parameters met acceptance criteria, confirming that the method is suitable for the accurate quantification of ST in rat plasma (Supplementary Materials).

### 2.6. Pharmacodynamic Evaluation of ST DPI in ALI

#### 2.6.1. In Vitro Anti-Inflammatory Analysis

To investigate the in vitro anti-inflammatory effect of ST on cells, MH-S cells stimulated by LPS were used as the inflammatory model to assess the suppressive impact of ST on the release of IL-6 and TNF-α. MH-S cells were incubated in a 12-well culture plate at a density of 2 × 10^5^ cells/well overnight, followed by co-treatment with 100 ng/mL LPS and series ST concentrations in RPMI 1640. At last, the supernatant was employed to investigate the release of IL-6 and TNF-α by ELISA.

#### 2.6.2. Pharmacodynamic Study

To explore the therapeutic efficacy of ST for ALI, an experimental test was conducted using 42 male SD rats weighing 250 ± 20 g. After 7 days of adapting feeding, the rats were randomly divided into seven groups: control, model, dexamethasone (DEX as positive drug, 3 mg/kg), SSA water extract (BJZ, 0.81 g/kg), and ST DPI low-, medium-, and high-dose groups (4, 8, and 16 mg/kg, calculated as ST). The dosage of BJZ was calculated by converting the recommended human dosage from the *Chinese Pharmacopoeia* 2020. The low, medium, and high doses of ST DPI were derived by integrating the recommended human dosage from the *Chinese Pharmacopoeia* 2020 with the actual extraction yield of ST purified based on preliminary experimental results. ALI models, except for the blank group, were induced by intratracheal LPS atomization (8 mg/kg) in isoflurane-anesthetized rats positioned supinely. Then, the rats were treated by ST and DEX 2 h later. The tissue samples were collected 6 h after the administration. ST DPI was administered intratracheally to rats using a Dry Powder Insufflator™-Model DP-4 (Penn-Century, Wyndmoor, PA, USA). Anesthesia was induced with Zoletil 50 at the dosage recommended by the manufacturer, and blood (4 mL) was collected from the abdominal aorta to isolate serum for the analysis of inflammatory cytokines (TNF-α, IL-6, and IL-1β). Lung tissues were harvested, with the middle lobe used for wet weight measurement, the right upper lobe fixed in 4% paraformaldehyde for HE staining, and the remaining lobes stored at −80 °C for the assessment of oxidative stress markers (SOD, MDA). Pathological scoring of lung HE sections was carried out according to the Smith scoring method [25]. Western blot (WB) was used to detect key proteins in lung tissue identified via network pharmacology, exploring the potential mechanisms of ST in ALI treatment.

## 3. Results and Discussion

### 3.1. Predicting Targets for ST in ALI Treatment via Network Pharmacology

#### 3.1.1. Target Prediction, Screening, and the PPI Network

**Figure 1:**
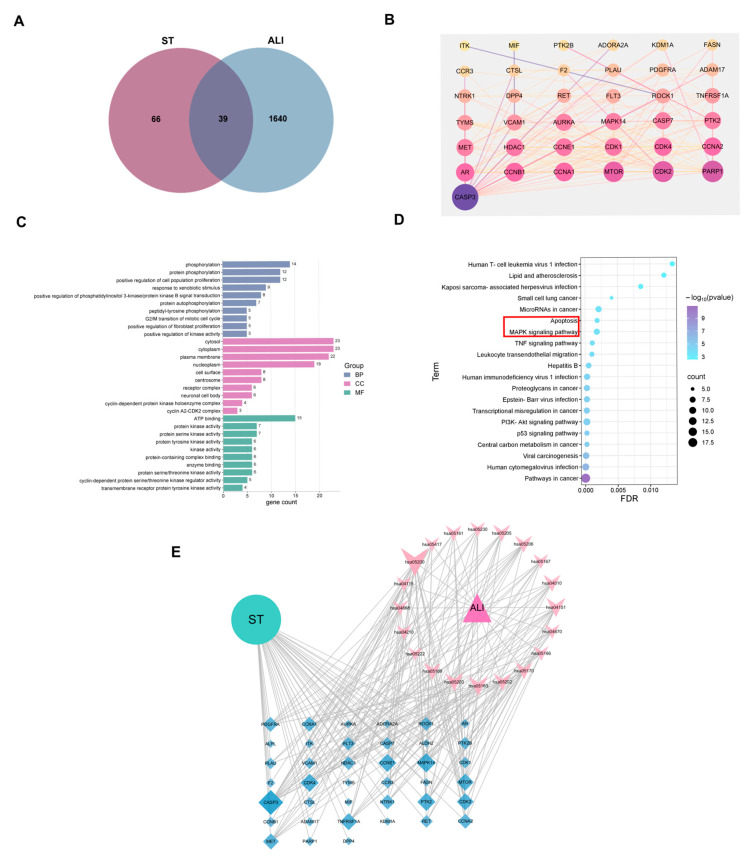
Analysis of the mechanism of ST in the treatment of ALI through network pharmacology. (**A**) The intersection genes of components and diseases. (**B**) Construction of PPI network. (**C**) GO enrichment analysis. (**D**) KEGG enrichment analysis (The red box indicated potential pathways identified through target screening). (**E**) Construction of ST–target–ALI pathway network.

The monomer ST contained 105 targets, and 1679 targets were associated with ALI disease, obtaining an intersection map with 39 common genes through the bioinformatics platform (Figure 1A). The 39 intersecting targets were analyzed using the STRING online platform to construct a PPI network, including 193 edges and 39 nodes, with an average node degree of 9.9 and an average local clustering coefficient of 0.266. The top-ranked potential core targets of ST for ALI treatment were identified as follows: CASP3, MAPK14, PTK2, CCNE1, CDK4, MTOR, CDK2, and TNFRSF1A (Figure 1B).

#### 3.1.2. GO Biological Function and KEGG Enrichment Analysis

The results of the GO biological accumulation analysis revealed 152 entries within the BP category, 36 entries in the CC category, and 71 entries in the MF category (Figure 1C). In the BP category, processes like phosphorylation, protein phosphorylation, the positive regulation of cell population proliferation, kinase activity regulation, and xenobiotic stimulus response were involved. Components such as cytosol, cytoplasm, plasma membrane, nucleoplasm, and cell surface were included for CC. In MF, functions like ATP binding, protein kinase, protein serine/threonine kinase, protein tyrosine kinase, and kinase activities were identified. Protein phosphorylation critically regulates inflammation regulation by precisely controlling the activity [26] and function of inflammatory cells through the coordinated actions of kinases, phosphatases, and mitochondrial stability [27]. Redox modification directly affects kinase activity by regulating the protein phosphorylation signaling pathway, thereby modulating cell growth, proliferation, and inflammatory responses [28]. In summary, ST may primarily treat ALI through anti-inflammatory and antioxidant effects. The relevant pathways were screened and an enrichment bubble diagram was created. In the construction of the ST–target–ALI pathway network, CASP3 and MAPK14 were identified as key targets associated with ALI-related inflammation. It was hypothesized that the therapeutic mechanism of ST in ALI treatment may involve the regulation of apoptosis through the MAPK signaling pathway (Figure 1D,E).

#### 3.1.3. Molecular Docking Simulation

The degree values of key components and disease-related targets were evaluated by constructing the ST–target–ALI pathway network. Based on the top-ranked targets and GO/KEGG analysis, CASP3 and MAPK14 were selected for molecular docking with ST and subsequent visualization. The active docking box was established based on the crystallographic position of the PDB site annotation of the core target protein: MAPK14 (center_x = 1.089, center_y = 20.284, center_z = 35.88) and CASP3 (center_x = 14.877, center_y = 6.779, center_z = 22.159). The docking results demonstrated that the core compound molecules of all drugs were stably bound within the docking box of the core target protein. The binding energy of ST to MAPK14 was −5.7 kcal·mol^−1^ and to CASP3 was −6.5 kcal·mol^−1^, which were small for stable binding. These two targets were associated with ALI, suggesting their significance in the treatment of ALI by ST (Supplementary Materials, Figure S2).

A network pharmacology analysis revealed the therapeutic potential of ST in addressing ALI. To leverage this activity, an inhalable dry powder formulation of ST was engineered for targeted pulmonary delivery, offering a strategic approach to enhance the therapeutic efficacy against ALI through localized respiratory administration.

### 3.2. Powder Characteristics of ST DPI

#### 3.2.1. Powder Characterization

**Figure 2:**
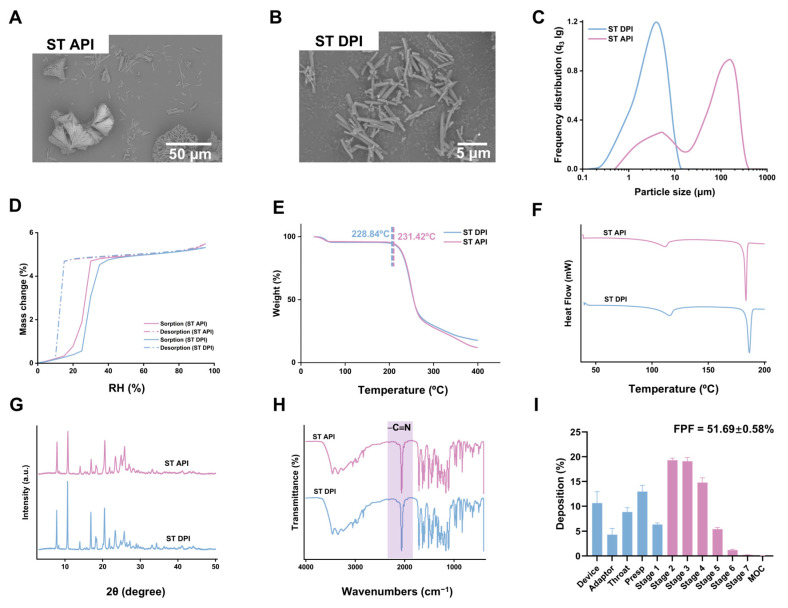
Characterization of ST API and ST DPI, along with the in vitro deposition distribution of ST DPI. SEM images of ST API (**A**) and ST DPI (**B**). Particle size distribution of ST API and ST DPI (**C**). Images of moisture adsorption curves (**D**), TGA curves (**E**), DSC thermal patterns (**F**), XRD pattern (**G**), FTIR spectrogram (**H**), and in vitro deposition distribution of ST DPI (**I**).

The morphology of ST DPI and API was investigated via SEM. The results revealed that the ST API consisted of needle-like crystals with an extended crystal length, while ST DPI transformed into a dispersed needle-like crystal with an approximate length of 5 μm after micronization (Figure 2A,B). The particle size of ST API measured by the laser diffraction showed an ununiform distribution with D_50_ at 65.17 µm and D_90_ at 204.64 µm and only 17.87% in the 1–5 μm range. After ultrasonic-assisted antisolvent micronization, the values of D_50_ and D_90_ were 2.99 μm and 6.93 μm, and the percentage of particles in the range of 1–5 μm reached 62.58% (Figure 2C). The calculated span was 2.04, which indicated a moderate distribution breadth in the ST DPI formulation. It is likely due to its needle-shaped crystal habit. The measurements were collected using laser diffraction (PAQXOS-HELOS|RODOS, Sympatec GmbH, Clausthal-Zellerfeld, Germany), reporting volume-based distributions calculated via the Fraunhofer model assuming spherical particles. For needle-shaped crystals, this spherical equivalence assumption can artificially broaden the apparent distribution. Additionally, such crystals are inherently more difficult to disperse and maintain in a dispersed state, which may contribute to the higher powder dispersion observed in ST DPI formulations.

The moisture absorption test showed a 5.1% weight increase in ST API at 80% RH, which exhibited certain hygroscopicity (Figure 2D). TGA was used to investigate the thermal stability of ST. The thermal decomposition of ST API and DPI occurred approximately at 230 °C, demonstrating its insensitivity to heat (Figure 2E). The DSC curve exhibited consistent melting peaks at approximately 114 °C and 185 °C for both ST API and ST DPI, respectively. Meanwhile, the XRD patterns of both ST API and ST DPI displayed similar diffraction peaks at identical positions, with distinct characteristic peaks observed at 2θ values of 7.8°, 10.6°, 13.9°, 16.9°, and 20.5°, which showed that the crystal form of ST remained unchanged after the antisolvent micronization (Figure 2F,G). The FTIR spectrum showed that the characteristic cyanogen peak was at 2060 cm^−1^, showing that the functional groups of ST remained unchanged after micronization (Figure 2H). In summary, the antisolvent-processed ST DPI demonstrated a favorable aerodynamic performance and robust crystalline stability.

#### 3.2.2. Aerodynamic Particle Size Distribution

The aerodynamic particle size distribution was evaluated by NGI to obtain the parameters of the ST DPI with the ED of 24.10 ± 1.06 mg, the FPD of 12.47 ± 0.47 mg, and the FPF of 51.69 ± 0.58%, which demonstrated that ST DPI exhibited a favorable lung deposition efficiency (Figure 2I). The MMAD was measured at 3.73 ± 0.05 μm and the GSD at 1.81 ± 0.02, representing a narrower particle size distribution (i.e., GSD closer to 1). The recovery rate was 96.22 ± 1.49%, which exhibited minimal loss with no rebound or secondary impact observed. The emptying rate was 98.85 ± 0.81%, indicating that ST DPI was almost entirely emitted. The DDU of 10 capsules fell between 75% and 125%. In all, the ST DPI exhibited favorable aerodynamic properties, achieving efficient lung delivery.

### 3.3. Pharmacokinetic Analysis

The pharmacokinetic results demonstrated that the pulmonary administration of ST significantly enhanced systemic absorption and improved bioavailability, likely attributed to the avoidance of the first-pass effect, while exhibiting triexponential pharmacokinetic characteristics comparable to those observed with intravenous injection. The prolonged MRT_0–∞_ of ST attributed to its unique pharmacokinetic properties supports the therapeutic feasibility of the ST DPI for ALI.

#### 3.3.1. Pharmacokinetics of ST Intravenous and Inhaled Administration

**Figure 3:**
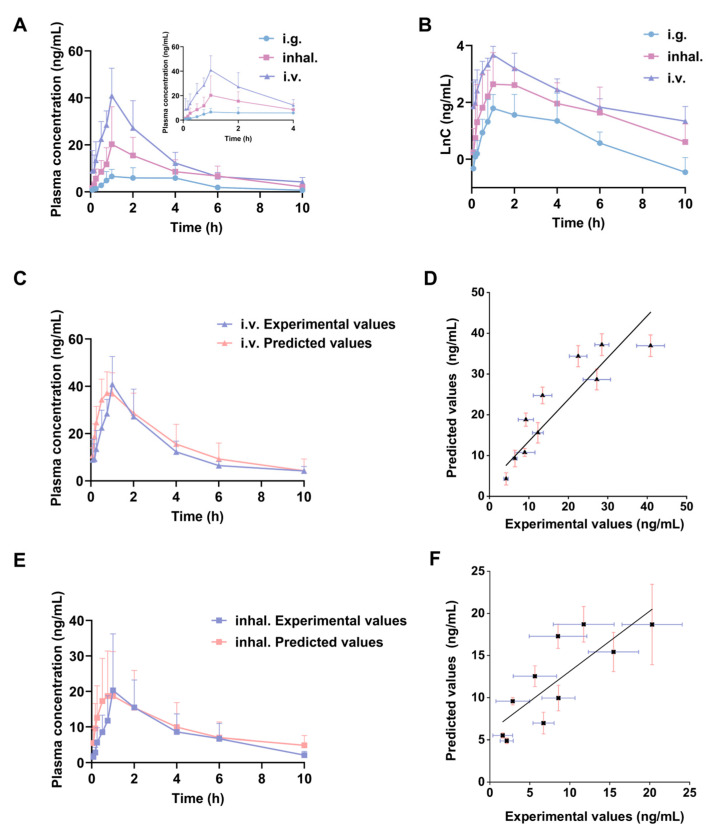
Pharmacokinetic research on ST DPI. (**A**) Mean plasma concentration–time profiles of ST in rats after intragastric, inhaled, and intravenous administrations. (**B**) LnC–time curves of ST in rats after intragastric, inhaled, and intravenous administrations (*n* = 6). (**C**) Experimental values and three-compartment model predicted values of ST i.v. plasma concentrations. (**D**) Error analysis of actual value and projected value of ST i.v. plasma concentrations. (**E**) Experimental values and three-compartment model predicted values of ST inhal. plasma concentrations. (**F**) Error analysis of actual value and projected value of ST inhal. plasma concentrations.

A pseudo-absorption phase was observed in the high-dose group (16 mg/kg) of ST following intravenous administration via the tail vein, prompting further investigation. Additionally, the logarithm of plasma drug concentration versus time (lnC–t) curve following ST caudal vein administration exhibited two distinct elimination phases. Similarly, the pulmonary inhalation group exhibited comparable absorption peak and pharmacokinetic trends, with its lnC–t curve also revealing characteristic biphasic elimination behavior (Figure 3A,B). This observation led to the hypothesis that upon intravenous and inhaled administration, the drug interacts with an initial binding mechanism, undergoes subsequent release, and is ultimately eliminated, resulting in three exponential phases characteristic of a three-compartment model. The plasma drug concentration data from each rat in the caudal vein injection and inhaled groups were analyzed using the two-stage residual method to establish a three-compartment model (Equation (2)).

C = Ae^−αt^ + Be^−βt^ + De^−γt^ (2)

The fitted ST intravenous three-compartment model was used to calculate predicted plasma drug concentrations for each rat in the caudal vein injection group, generating corresponding concentration–time profiles (Table 1). The error analysis between the measured and predicted values (R^2^ = 0.887) confirmed the three-compartment pharmacokinetic characteristics of ST following caudal vein injection in rats. Using the same methodology, the pharmacokinetic analysis of ST DPI revealed a moderate correlation between the predicted and observed plasma concentrations (R^2^ = 0.611) (Figure 3C–F).

#### 3.3.2. Pharmacokinetic Parameters of ST

The pharmacokinetic results demonstrated that ST inhalation administration significantly increased the AUC compared with oral gavage (*p* < 0.05). The absolute bioavailability of the ST DPI was 79.12% relative to intravenous administration, while oral gavage exhibited an absolute bioavailability of 29.82%. Notably, the pulmonary inhalation of ST demonstrated a 2.7-fold enhancement in bioavailability compared with intragastric administration. Compared with the ST intragastric group, the C_max_ of the ST inhaled administration group was approximately 2.5 times higher, and the peak time T_max_ occurred 0.34 h earlier (Table 2).

On the other hand, the pharmacokinetic data revealed a prolonged MRT_0–∞_ for ST, contrasting with the rapid onset typically observed with conventional DPIs [29,30]. This distinct behavior may be associated with its three-compartment model pharmacokinetic characteristics. The comparable pharmacokinetic profiles between inhaled and intravenous administration suggested that inhaled ST enhanced pulmonary drug deposition while prolonging systemic retention time, thereby potentiating therapeutic efficacy in pulmonary diseases.

#### 3.3.3. Discussion of ST Pseudo-Absorption Peak

In the present study, the inhaled and intravenous administration of ST at the higher dose of 16 mg/kg led to a pseudo-absorption peak in the plasma concentration–time profile, with pharmacokinetic behavior described by a three-compartment model. However, the literature has reported the absence of pseudo-absorption following the administration of a lower dose (2 mg/kg) via intravenous administration [8]. These findings suggested that the dose may influence the pharmacokinetic profile of ST. Moreover, significantly prolonged MRT and t_1/2_ were observed at the higher dose (16 mg/kg) compared with literature values for the lower dose (2 mg/kg). This indicates extended in vivo retention at the higher dose, which may prolong its pharmacological effect duration and enhance therapeutic efficacy.

The unique pharmacokinetics of ST potentially might be related to enterohepatic recycling (EHR). EHR refers to the process by which drugs are secreted from the liver into the bile, subsequently transported to the small intestine, reabsorbed by enterocytes, and ultimately returned to the liver [31]. It is commonly associated with oral drugs, while it is also considered as EHR if multiple plasma concentration peaks occurred following intravenous administration [32,33]. ST exhibits poor oral bioavailability and is primarily excreted in feces. However, the concentration–time profile of ST following inhaled and intravenous administration lacked multi-peaking, instead featuring a singular pseudo absorption. Therefore, whether the unique pharmacokinetics of ST involve EHR requires further investigation. This could be elucidated by the measurement of ST concentrations in bile following intravenous administration to rats.

An alternative possibility was that the initial rapid distribution of high-dose ST into the bloodstream led to its accumulation in tissues [34]. Upon the saturation of tissue concentrations, the concentration gradient reversed, allowing drug re-entry into the bloodstream and resulting in an apparent absorption phase. The apparent volume of distribution (V) is a theoretical parameter characterizing drug distribution. A higher V value typically indicates a greater propensity for tissue accumulation [35,36]. In rats, the total volume of distribution constitutes approximately 60–70% of body weight (0.6–0.7 L/kg). The markedly high terminal volume of distribution (Vz, inhal. = 1205 ± 887.4 L/kg; Vz, i.v. = 405.7 ± 185.9 L/kg) observed following ST administration via inhaled and intravenous administration suggested a high affinity or binding to tissue components, leading to tissue “storage”. This mechanism likely gives rise to the observed pseudo-absorption peak. The specific sites of tissue distribution remain to be elucidated through dedicated tissue distribution studies.

### 3.4. Pharmacodynamics of ST in the Treatment of ALI

#### 3.4.1. Anti-Inflammatory Effects of ST at the Cellular Level

**Figure 4:**
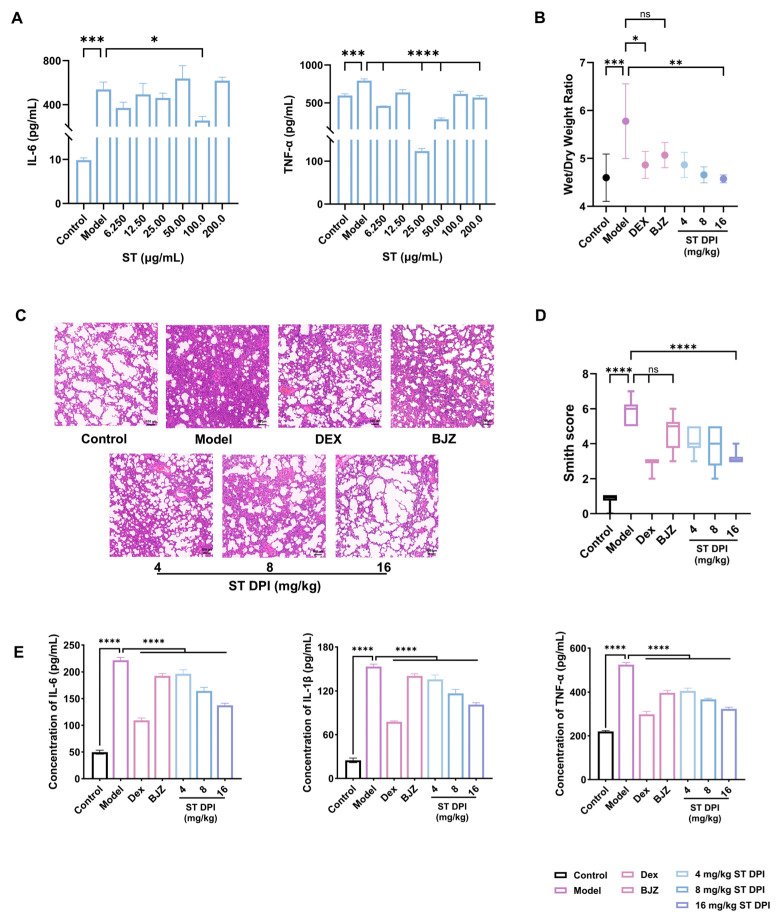
Pharmacodynamic evaluation of ST DPI. (**A**) Inhibitory effect of ST on inflammatory cytokines IL-6 and TNF-α at the cellular level. (**B**) Wet–dry weight ratio of the middle lobe of the lung. (**C**) Histopathological examination of HE-stained ALI rat lung tissues (100×, scale plate = 100 μm). (**D**) Smith score of lung pathological section. (**E**) Expression levels of inflammatory factors IL-6, IL-1β, and TNF-α in ALI rat serum (**** *p* < 0.0001, *** *p* < 0.001, ** *p* < 0.01, * *p* < 0.05, ns ≥ 0.05).

LPS-stimulated MH-S cells were utilized as an inflammation model to evaluate the inhibitory effect of ST on the release of IL-6 and TNF-α within the safe dose range. The results demonstrated that ST exhibited the strongest suppression of IL-6 at 100 μg/mL and TNF-α at 25 μg/mL. ST exhibited a certain inhibitory effect on LPS-induced inflammation at the cellular level (Figure 4A).

Given that the cytotoxicity assay established the maximum safe concentration of ST for MH-S cells as 100 μg/mL (Figure S4), it was postulated that the elevation in IL-6 content at this concentration was likely related to the cytotoxicity of ST. The maximal inhibitory effect of ST on TNF-α was observed at 25 μg/mL. Notably, ST failed to effectively reduce TNF-α levels at higher concentrations. This phenomenon is potentially attributable to the intrinsic property of ST to promote TNF-α release [37], thereby counteracting its putative inhibitory effect in the cell-based anti-inflammatory assay.

#### 3.4.2. Lung Wet–Dry Weight Ratio and Lung Histopathological Observation and Score

The HE staining of rat lung tissues showed a normal architecture in healthy controls. In contrast, the ALI model group exhibited characteristic pathological features, including alveolar epithelial damage, interstitial and alveolar edema, inflammatory cell infiltration, severe protein exudation, alveolar wall thickening, and diffuse pulmonary edema, confirming successful ALI induction. In the ST DPI (16 mg/kg) group, the infiltration, congestion, and edema of inflammatory cells in the alveoli and interstitial lung were significantly improved. After treatment with DEX, BJZ, and low, medium, and high dosages of the ST DPI, the pulmonary inflammatory state manifested a discernible improvement. Combined with the lung wet-to-dry weight ratio and HE pathological sections, BJZ had a relatively poor effect, while the ST DPI indicated a dose-dependent improvement. The high-dose ST DPI group demonstrated a comparable effect to the DEX group (Figure 4B–D). The ST DPI demonstrated significant therapeutic efficacy for ALI at the pulmonary pathological level.

#### 3.4.3. Levels of Inflammatory Factors and Oxidative Factors

**Figure 5:**
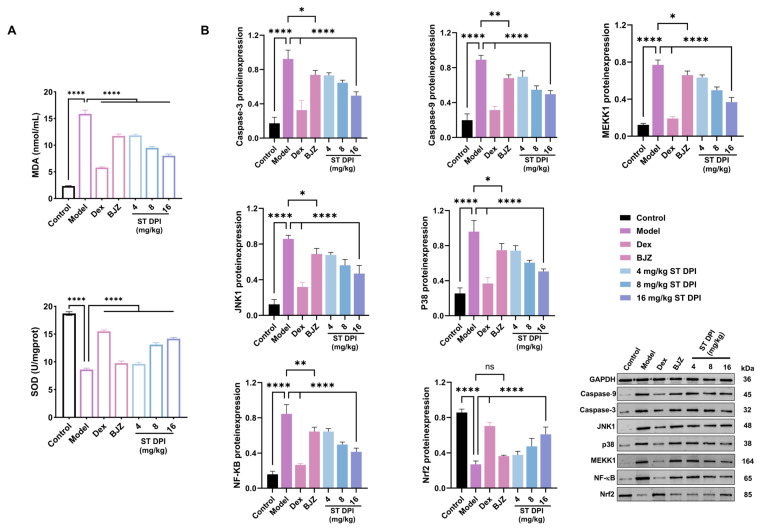
Mechanism of ST in treating ALI rats. (**A**) Antioxidant stress levels detected in the lungs of ALI rats. (**B**) Quantitative results of protein expression. (**** *p* < 0.0001, ** *p* < 0.01, * *p* < 0.05, ns ≥ 0.05).

As mentioned earlier, inflammatory factors and oxidative stress play a central role in ALI, mutually reinforcing each other and collectively contributing to lung tissue injury and dysfunction. Regarding the inflammation level, the ST DPI could decrease IL-6, IL-1β, and TNF-α in a dose-dependent manner (Figure 4E). In terms of oxidative stress, the level of MDA decreased and the level of SOD increased in a dose-dependent manner (Figure 5A). Overall, the ST DPI inhibited the occurrence of ALI by suppressing inflammatory factors and modulating oxidative stress. The therapeutic effects of the ST DPI were dose-dependent and superior to traditional SSA water extract (BJZ), and the high-dose ST DPI exhibited therapeutic efficacy approaching that of DEX.

#### 3.4.4. WB Validates the Mechanism of ST in ALI: The Positive Feedback of the MAPK Pathway on Cell Apoptosis

Based on the aforementioned research, the mechanisms of the ST DPI in treating ALI were further investigated in conjunction with network pharmacology results. MAPKs regulate apoptosis via transcriptional and post-transcriptional mechanisms, with p38 and JNK activation generally exerting pro-apoptotic roles. Caspase-3 enhances apoptosis through a positive feedback loop by directly activating Caspase-9, while also initiating MEKK1 activation. MEKK1 further amplifies the apoptotic signal by inducing cytochrome C release and Caspase-9/-3 activation via JNK/p38 pathways [38,39,40]. WB analysis confirmed the mechanism of ST in ALI treatment, aligning with prior network pharmacology findings, demonstrating its regulation of apoptosis via the MAPK pathway. ST suppressed MEKK1 expression by inhibiting Caspase-9/3, ultimately reducing JNK/p38 activation, downregulating inflammatory protein expression and attenuating inflammation (Figure 5B).

The pathogenesis of ALI involves a self-perpetuating cycle of inflammation and oxidative stress. Central to this process, NF-κB drives the transcriptional activation of pro-inflammatory mediators (such as TNF-α, IL-1β, and IL-6), while Nrf2 counteracts oxidative damage by upregulating antioxidant enzymes [41,42]. The p38 MAPK pathway plays a dual role in this process: while it enhances ROS production, activating NF-κB and amplifying inflammation, it also inhibits Nrf2 nuclear translocation by phosphorylation, thereby suppressing antioxidant responses and exacerbating oxidative stress injury [43]. This interplay between NF-κB, Nrf2, and p38 MAPK underscores their critical roles in balancing inflammation and oxidative stress, with dysregulation contributing to disease progression. The WB analysis confirmed that ST reduced the activation of NF-κB in the MAPK positive feedback loop, promoted the nuclear translocation of Nrf2, and alleviated inflammation and oxidative damage.

In summary, the WB results indicated that compared with the model group the aqueous extract of SSA had a weaker ability to inhibit the positive cycle of cell apoptosis. Conversely, ST DPI displayed a dose-dependent inhibitory effect on the expression of related proteins (Figure 5B). Notably, the effect of high-dose ST DPI was comparable to that of DEX. The potential mechanism of ST in treating ALI involved the inhibition of MAPK-mediated positive feedback apoptosis and the suppression of NF-κB and Nrf2 expression.

## 4. Conclusions

This study establishes the ST DPI as an innovative therapeutic strategy for ALI through its unique triexponential pharmacokinetic profile and multimodal mechanism of action. The developed pulmonary delivery system achieved efficient alveolar deposition, coupled with three-compartment model pharmacokinetics that enhance drug bioavailability while maintaining sustained therapeutic exposure. The ST DPI demonstrates prolonged pulmonary retention characteristics, which provide therapeutic advantages for treating respiratory disorders. Beyond its pharmacokinetic advantages, the ST DPI exerts comprehensive therapeutic effects by simultaneously modulating apoptotic processes via MAPK pathway regulation and suppressing pivotal inflammatory signaling cascades. The ST DPI demonstrated significant anti-inflammatory and antioxidant effects in a rat model of ALI. The mechanisms underlying triexponential pharmacokinetics with high-dose ST (inhalation/intravenous) require further elucidation. Overall, ST was formulated as a novel pulmonary delivery system; the convergence of targeted pulmonary delivery with peculiar pharmacokinetics offers a promising approach to address ALI.

## Figures and Tables

**Table 1:** Parameters of the three-compartment model for each rat in the intravenous and inhaled groups (mean ± SD, *n* = 6).

Parameters	α	β	γ	A	B	D
Intravenous	2.92 ± 0.88	0.39 ± 0.23	0.12 ± 0.09	−70.71 ± 41.48	4.57 ± 182.80	66.14 ± 169.74
Inhaled	3.02 ± 0.70	0.11 ± 0.29	0.23 ± 0.25	−26.43 ± 15.64	12.40 ± 15.25	14.04 ± 18.10

**Table 2:** Pharmacokinetic parameters of ST (16 mg/kg) after intragastric, inhaled, and intravenous administrations (Mean ± SD, *n* = 6).

Parameter	Intragastric	Inhaled	Intravenous
AUC_0–t_ (μg/L·h)	41.44 ± 23.86	111.7 ± 63.42	135.5 ± 38.37
AUC_0–∞_ (μg/L·h)	43.27 ± 23.62	114.8 ± 62.98	145.1 ± 41.28
MRT_0–t_ (h)	5.574 ± 0.796	6.386 ± 1.799	3.033 ± 0.267
MRT_0–∞_ (h)	7.273 ± 2.044	8.03 ± 2.859	4.105 ± 0.615
t_1/2_ (h)	4.830 ± 2.337	4.433 ± 1.391	2.345 ± 0.684
T_max_ (h)	2.167 ± 1.472	1.833 ± 1.169	0.958 ± 0.102
C_max_ (μg/L)	8.74 ± 5.359	21.48 ± 15.02	41.30 ± 11.12

## Data Availability

The data is not publicly available at this time as it will be used in other ongoing studies.

## Supplementary Materials

The following supporting information can be downloaded at: https://www.mdpi.com/article/10.3390/pharmaceutics17070909/s1, Figure S1. Identification of ST purified product and investigation of micronization process. (A) HPLC chromatogram of ST purified product and ST standard. (B) ^1^H-NMR chromatogram of ST purified product and ST standard. (C) The influence of the size of magnetic rotor and ultrasound on the particle size of antisolvent (dashed line shows the left-axis cumulative distribution and solid line indicates the right-axis frequency distribution); Table S1. ST API particle size and post-micronization size; Table S2. Compound–target correlation and binding value stats; Figure S2. Visualization of molecular docking. (A) Visualization of ST docking with CASP3. (B) Visualization of ST docking with MAPK14; Figure S3. Full scan mass spectrum of ST in positive ion mode; Figure S4. HPLC-MS/MS specific chromatogram of ST in rat plasma. (A) Blank plasma; (B) Blank plasma with IS; (C) Blank plasma with ST and IS; Figure S5. Cell viability of ST DPI against MH-S cells; Table S3. HPLC−MS/MS methodology validation (*n* = 6); Table S4. The results of dilution reliability for the determination method of ST in rat plasma (*n* = 6). References [44,45] are cited in the Supplementary Materials.

## Abbreviations

The following abbreviations are used in this manuscript:

ALI	Acute lung injury
ST	Sinapine thiocyanate
DPI	Dry powder inhaler
SSA	Sinapis Semen Albae
LPS	Lipopolysaccharides
PBS	Phosphate-buffered saline
IL-1β	Interleukin-1β
IL-6	Interleukin-6
TNF-α	Tumor necrosis factor-α
NF-κB	Nuclear factor kappa-B
Nrf2	Nuclear factor erythroid 2-related factor 2
JNK1	C-Jun N-terminal kinase 1
MEKK1	MAPK kinase kinase 1
p38	p38 mitogen-activated protein kinase
GAPDH	Glyceraldehyde-3-phosphate dehydrogenase
IACUC	Institutional Animal Care and Use Committee
PPI	Protein–protein interaction
BP	Biological process
CC	Cellular component
MF	Molecular function
KEGG	Kyoto Encyclopedia of Genes and Genomes
GO	Gene Ontology
SEM	Scanning electron microscopy
TGA	Thermal gravimetric analyzer
DSC	Differential scanning calorimetry
DVS	Dynamic moisture adsorption
XRD	X-ray diffractometer
FTIR	Fourier transform infrared
FPD	Fine particle dose
ED	Emitted dose
FPF	Fine particle fraction
MMAD	Median aerodynamic diameter
GSD	Geometric standard deviation
DDU	Delivered dose uniformity
WB	Western blot
