# Supplementary material for: Three-Compartment Pharmacokinetics of Inhaled and Injected Sinapine Thiocyanate Manifest Prolonged Retention and Its Therapeutics in Acute Lung Injury
Source: Pharmaceutics. 2025 Jul 14;17(7):909. doi: 10.3390/pharmaceutics17070909 (PMC12300973; doi:10.3390/pharmaceutics17070909)
Supplement: Supplementary file 1 [file pharmaceutics-17-00909-s001.zip › pharmaceutics-3690026-supplementary.pdf]

pharmaceutics

MDPI

Article

Three-Compartment Pharmacokinetics of Inhaled and Injected Sinapine Thiocyanate Manifest Prolonged Retention and Its Therapeutics in Acute Lung Injury

Zixin Li [1,2,3,†], Caifen Wang [2,4,†], Huipeng Xu [2], Qian Wu [2,3,4], Ningning Peng [2,3,4], Lu Zhang [5], Hui Wang [5], Li Wu [2], Zegeng Li [6], Qinjun Yang [5,*] and Jiwen Zhang [1,2,3,4,*]

[1] College of Pharmacy, Anhui University of Chinese Medicine, Hefei 230012, China; zixinli201475@163.com
[2] Center for Drug Delivery Systems, Shanghai Institute of Materia Medica, Chinese Academy of Sciences, Shanghai 201210, China; wangcaifen@simm.ac.cn (C.W.); hpxu713@163.com (H.X.); 19834519869@163.com (Q.W.); pnn19863823375@163.com (N.P.); wuli@simm.ac.cn (L.W.)
[3] Jiangsu Yungou Pharmaceutical Technology Co., Ltd., Yangtze Delta Drug Advanced Research Institute, Nantong 226133, China
[4] College of Pharmacy, Shenyang Pharmaceutical University, Shenyang 110016, China
[5] College of Chinese Medicine, Anhui University of Chinese Medicine, Hefei 230038, China; 18715028922@163.com (L.Z.); 15077916827@163.com (H.W.)
[6] Department of Respiratory, The First Affiliated Hospital of Anhui University of Chinese Medicine, Hefei 230031, China; li6609@126.com
* Correspondence: yangqinjun@ahtcm.edu.cn (Q.Y.); jwzhang@simm.ac.cn (J.Z.)
† These authors contributed equally to this work.

1. Extraction of sinapine thiocyanate from sinapis semen albae and development of a dry powder inhalation formulation

1.1. Materials and methods

1.1.1. Materials

SSA was supplied by Bozhou Hengyi Traditional Chinese Medicine Technology Co., Ltd. (Anhui, China), and the ST reference standard was supplied by Sichuan Cuiyirun Biotechnology Co., Ltd. (Sichuan, China). Ethanol and methanol were procured from Sinopharm Chemical Reagent Co., Ltd. (Shanghai, China), ethyl acetate, HPLC-grade methanol, and acetonitrile were supplied from Meryer Chemical Technology Co., Ltd. (Shanghai, China).

1.1.2. Extraction and purification of ST

ST was extracted, separated and purified from Sinapis Semen Albae (SSA). SSA was crushed and sieved through a 24-mesh sieve followed by degreasing with petroleum ether (1:6 liquid-to-solid ratio). The defatted SSA was reflux−extracted with 80% ethanol (1:15 liquid-to-solid ratio) for 4.5 h. The extract was concentrated using rotary evaporation, diluted with water and filtered. An equal volume of 20% potassium thiocyanate solution was added and then incubated at 4 °C for 10 h to precipitate ST crude crystals. The crystals were separated and redissolved in hot 95% ethanol. Recrystallization was performed by cooling to room temperature and storing at −20 °C for 4 h to obtain the purified ST product.

1.1.3. Identification of the components and purity of extract ST

The SSA extract was identified by qualitative analysis, including high-performance liquid chromatography (HPLC, Agilent, Santa Clara, CA, USA) and ^{1}H-Nuclear magnetic resonance (^{1}H-NMR, Bruker, Ettlingen, Germany). ST was carried on Spursil C18 column

 https://doi.org/10.3390/pharmaceutics17070909

(250 mm × 4.6 mm, 5 μm) at 30 °C and the mobile phase was acetonitrile and 0.02 mol/L potassium dihydrogen phosphate solution in a volume ratio of 15:85 (*v*/*v*) at a flow rate of 1.0 mL/min, with the wavelength of the UV (DAD) of 326 nm and the injection volume of 10 μL. ^{1}H-NMR identified the ST standard and ST purified product, which deuterated DMSO was used as a solvent, and the spectra were collected in a magnetic field at 400 MHz.

1.1.4. Ultrasonic-assisted antisolvent preparation of ST DPI

ST DPI was prepared via an ultrasound-assisted anti-solvent method utilizing methanol/ethyl acetate (1:10 *v*/*v*) with supersaturation–driven crystallization. Under the aforementioned conditions, the effects of small magnetic rotors (7 × 21 mm, formulation 1) and large magnetic rotors (8 × 30 mm, formulation 2) on the crystalline particle size of ST DPI were investigated. Furthermore, an ultrasonication-assisted optimized antisolvent crystallization method was incorporated for process enhancement (formulation 3). Finally, ST DPI was obtained through suction-filtration and drying at 60 °C for 4 h.

In all, an ultrasound-assisted anti-solvent method was employed to prepare ST DPI, which leveraged the principles of solubility differences and supersaturation-driven crystallization to control crystal particle size. Ultrasound effectively improved the aspect ratio of needle-shaped ST crystals by disrupting them along the long axis or promoting the growth of the slowest-growing surfaces, making it suitable for the preparation of DPI.

1.1.5. Examination of residual solvents in ST DPI

Chromatographic separation was achieved using a DB-624 capillary column (30.0 m × 0.53 mm × 3.00 μm) with high–purity nitrogen as the carrier gas at a flow rate of 3.0 mL/min. The split injection mode was set at a ratio of 5:1, and the injector temperature was maintained at 250°C. The oven temperature program initiated at 45°C (5 min hold), followed by a 5°C/min ramp to 70°C (5 min final hold). Detection was performed by a flame ionization detector (FID) operated at 250°C. Headspace parameters included a 30 min equilibration at 80°C, with the sampling loop and transfer line temperatures set at 110°C and 120°C, respectively, using a 1 mL injection volume.

1.2. Results

1.2.1. HPLC and ^{1}H-NMR of ST extraction

HPLC analysis demonstrated that the chromatographic peak of the purified ST product exhibited a retention time (4.7 min) and peak profile identical to the ST standard when analyzed under Chromatographic Method 1.1.3 (Figure S1A). A single predominant peak was observed at this retention time. The purity of extract ST was calculated to be approximately 97.02%.

The ^{1}H NMR spectrum of the purified ST product revealed that its chemical shifts (δ), coupling constants (*J*), and proton signal integration ratios closely matched those of the reference standard under identical analytical conditions.

For ST-standard: ^{1}H NMR (400 MHz, DMSO-d_6) δ 9.04 (s, 1H, H-9), 7.62 (d, *J* = 15.9 Hz, 1H, H-10), 7.04 (s, 2H, H-4,6), 6.56 (d, *J* = 15.9 Hz, 1H, H-11), 4.59 (t, *J* = 5.0 Hz, 2H, H-13), 3.81 (s, 6H, H-7,8), 3.76 - 3.69 (m, 2H, H-14), 3.18 (s, 9H, H-15,16,17).

For ST-sample:^{1}H NMR (400 MHz, DMSO-d_6) δ 9.04 (s, 1H, H-9), 7.61 (d, *J* = 15.9 Hz, 1H, H-10), 7.03 (s, 2H, H-4,6), 6.55 (d, *J* = 15.9 Hz, 1H, H-11), 4.58 (d, *J* = 5.1 Hz, 2H, H-13), 3.81 (s, 6H, H-7,8), 3.74 - 3.67 (m, 2H, H-14), 3.17 (s, 9H, H-15,16,17).

The hydrogen atom at δ 9.05 was labile, exhibited randomness in its spectral signal, which was not distinctly observable in the reference standard but could be detected upon magnification. The signal at 2.5 ppm is assigned to the deuterated DMSO solvent, while the peak observed at 3.34 ppm corresponds to residual water in deuterated DMSO[1,2].

In all, the HPLC and ^{1}H-NMR spectra confirmed that the extracted substance from SSA was ST.

1.2.2. Optimization of anti–solvent process

The anti–solvent method was used to prepare ST DPI and revealed that the size of the magnetic rotor and the application of ultrasonication were critical for the particle size distribution of the powder significantly. Experimental results demonstrated that ST DPI prepared with a small magnetic rotor (7 × 21 mm, formulation 1) exhibited a more uniform particle size distribution (span = 2.80), whereas ST DPI prepared with a large magnetic rotor (8 × 30 mm, formulation 2) showed a broader particle size distribution (span = 3.64). The ST raw material particles had needle-like crystal morphology and the length of a single crystal could be controlled by stirring speed and ultrasonic during the anti-solvent process. Ultrasonic-assisted treatment was introduced to further optimize particle size (formulation 3), effectively disrupting needle–like crystals along their vertical axis, generating smaller and more uniformly distributed particles (Figure S1C and Table S1).

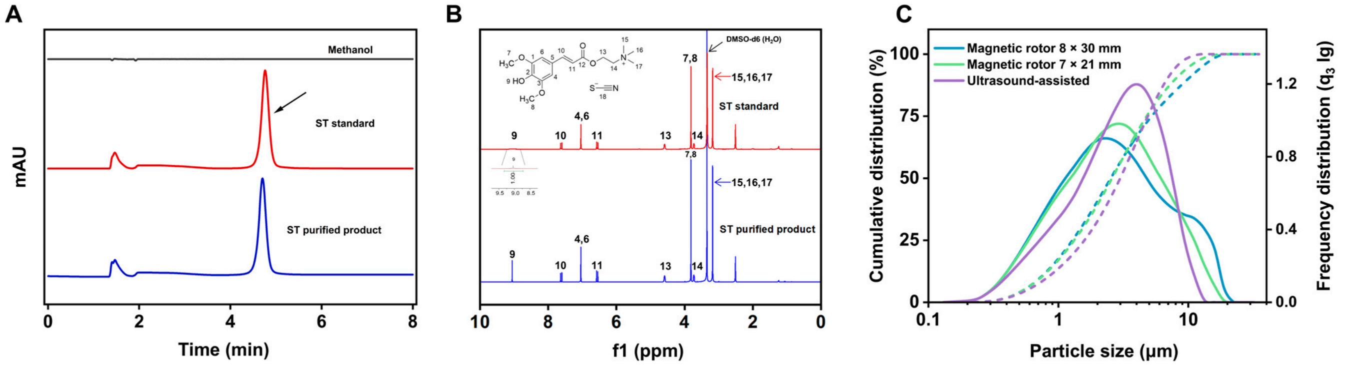

Figure S1. Identification of ST purified product and investigation of micronization process. (**A**) HPLC chromatogram of ST purified product and ST standard. (**B**) ^{1}H–NMR chromatogram of ST purified product and ST standard. (**C**) The influence of the size of magnetic rotor and ultrasound on the particle size of antisolvent (The dashed line showed the left–axis cumulative distribution and the solid line indicates the right–axis frequency distribution).

Table S1. ST API particle size and post-micronization size

Group	D_{10} (μm)	D_{50} (μm)	D_{90} (μm)	1–5 μm ratio (%)
ST API	2.61	65.17	204.6	17.87
Formulation 1	0.73	2.54	9.98	56.19
Formulation 2	0.74	2.60	8.02	59.54
Formulation 3	0.76	2.74	6.33	65.69

1.2.3. Residual Solvent Determination for ST DPI

No methanol residue was detected in the ST DPI, while the residual ethyl acetate content was quantified as 0.37% ± 0.03%, complying with the established acceptance criteria for residual solvents.

1.3. Conclusion

The ST API was synthesized via hot alcohol reflux followed by recrystallization, with its purity confirmed to be 97.02% through HPLC and ^{1}H NMR spectroscopy. Subsequently, the ST DPI was prepared via an ultrasonication–assisted antisolvent crystallization method for further experimental investigations.

2. Visualization of sinapine thiocyanate docking with MAPK14 and CASP3 molecules

Format files using OpenBabel. Perform molecular docking with multiTomulti and record binding energies. Export the energies and select the lowest ones. A negative binding free energy (ΔG = −RTlnK) means system stability, with lower energy indicating more excellent stability. Visualize well−paired component−target combinations using PyMOL. The display orientation of ligand ST and the receptor was adjusted to optimally visualize the critical hydrogen bonding interactions.

Judging from the degree value, the top-ranked targets of ST acting on ALI are CASP3 and MAPK14. These key MAPK pathway proteins in apoptosis regulation are related to ALI inflammation and oxidative stress, so they were chosen for docking and visualization analysis.

Table S2. Compound−target correlation and binding value stats

Target	Degree	ΔG (Binding with ST)
CASP3	16	−5.7
MAPK14	11	−6.5

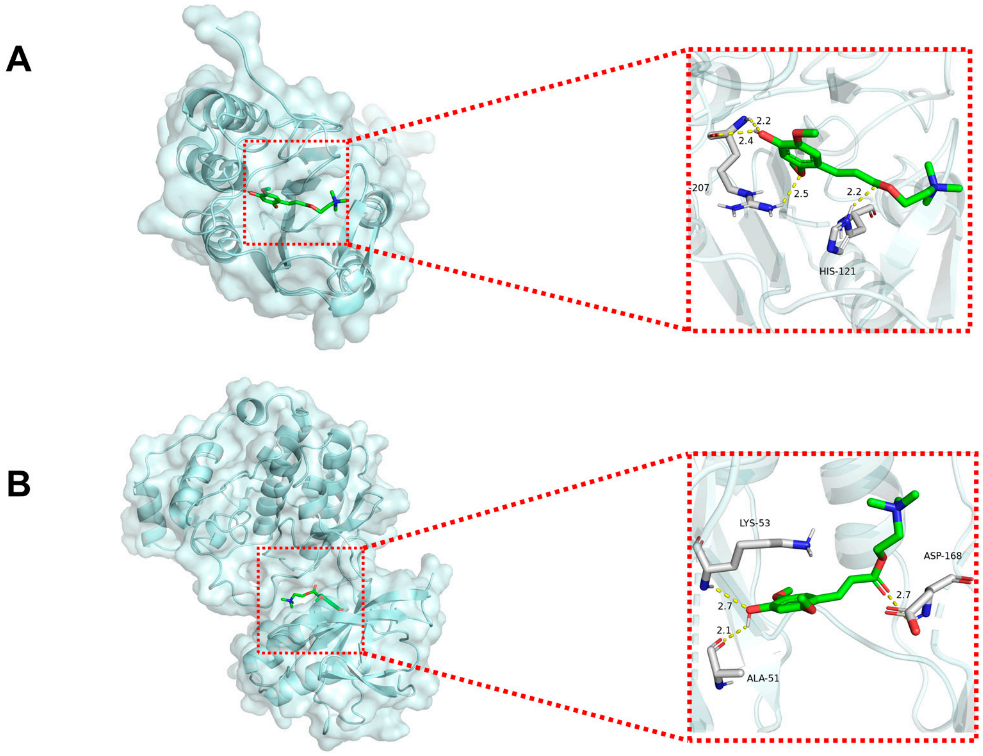

Figure S2. Visualization of molecular docking. (**A**) Visualization of ST docking with CASP3. (**B**) Visualization of ST docking with MAPK14.

3. HPLC-MS/MS methodology validation

3.1 *MS spectrum of ST*

HPLC-MS/MS (positive ESI mode): Full scan m/z 100–1000; ST MRM transition m/z 310.1 → 251.0.

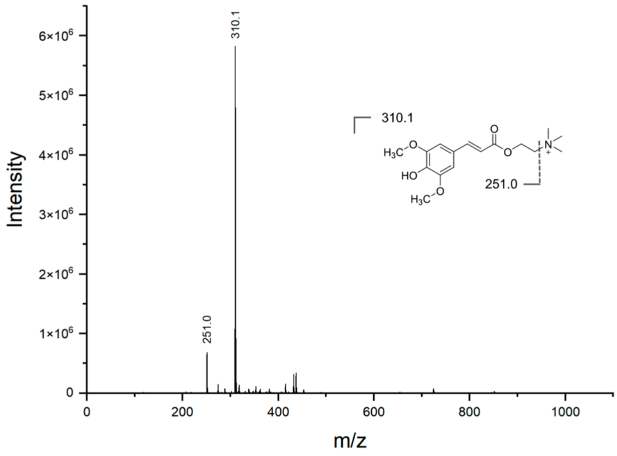

Figure S3. Full scan mass spectrum of ST in positive ion mode.

3.2 *Selectivity*

Selectivity of the method was determined by comparing the chromatograms of blank plasma samples from six different batches with the standard plasma samples spiked with ST and IS to check the potential interferences at retention times close to the analytes and IS. (Figure S3)

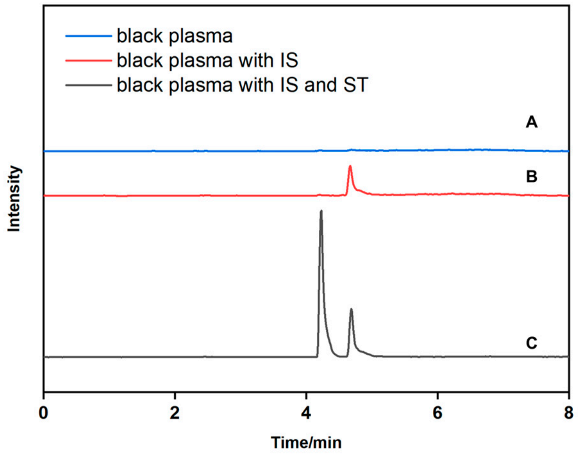

Figure S4. HPLC-MS/MS specific chromatogram of ST in rat plasma. (**A**) Blank plasma; (**B**) Blank plasma with IS; (**C**) Blank plasma with ST and IS.

3.3 *Calibration curves and lower limit of quantification*

Calibration curves were generated by plotting the peak area ratio of the analytes to IS (y) against the theoretical concentration (x) using a 1/x2 weighted least squares linear regression model. The regression equation was y = 27.53x + 0.1314. The lower limit of quantification (LLOQ) was defined as the lowest plasma concentra tion that could be quantified with acceptable precision and accuracy. For acceptable results, the correlation coeffi cient (r) of the calibration curve should be ≥0.99 and each back calculated

concentration has to be within ±15% of the nominal value, except at LLOQ, where deviation is acceptable up to ±20%.

3.4 Calibration curves and lower limit of quantification

Precision (RSD) and accuracy (RE) were evaluated by analyzing QCs at LLOQ in addition to three other concentration levels (low, medium and high) in six replicates on the same day or on three consecutive days. Acceptable criteria for precision were set at within ±15% RSD and accuracy below ±15% RE, except for the low concentration, where the value of the variation was allowed to be up to 20% (Table S3).

3.5 Recovery and matrix effect

The recovery and matrix effect were estimated in six replicates at three QC concentration levels. The extraction recoveries of the analytes were assessed by comparing the responses of the analytes extracted from QC samples with the responses of the analytes spiked in post-extraction samples. The matrix effects were determined by comparing the peak areas of the analytes in post-extraction samples with those of the analytes in neat samples at equivalent concentrations. Matrix effects and recoveries both showed RSD values within ±15%, meeting validation criteria (Table S3).

3.6 Stability

The stability of ST in rat plasma was investigated by analyzing low, medium and high level QC samples which were kept at room temperature for 24h, and −80°C after three freeze–thaw cycles (freeze-thaw stability). RSD was within ±15% and RE was within ±15% of the nominal values at all QC levels, meeting the acceptance criteria (Table S3).

3.7 Dilution reliability

The dilution reliability demonstrated by adding ST solution above the upper limit of quantification concentration to blank plasma, diluting 10 times with the same blank plasma as the sample to be tested, and then preparing 6 times in parallel. The accuracy should be accuracy below ±15% RE with a precision of less than 20% (Table S4).

Table S3. HPLC−MS/MS methodology validation (*n* =6).

Sample	Concentration (ng/mL)	Accuracy & Precision		Extraction recovery rate		Matrix effect		Stability			
								Room temperature		Freeze−thaw	
		RE (%)	RSD (%)	Mean (%)	RSD (%)	Mean (%)	RSD (%)	RE (%)	RSD (%)	RE (%)	RSD (%)
Plasma	0.20	11.16	5.30	85.00	5.30	114.94	11.1	13.73	8.92	7.16	3.57
	0.80	−4.63	4.40	91.48	4.40	108.59	6.05	2.64	5.15	7.05	2.54
	8.00	7.08	2.99	94.34	2.99	113.67	6.60	7.98	2.49	9.94	2.37

Table S4. The results of dilution reliability for the determination method of ST in rat plasma (*n* = 6)

C (ng/mL)	Measured (ng/mL)	RE (%)	RSD (%)
4.00	4.184	4.60	4.07
	3.8541	−3.65	
	4.1903	4.76	
	3.8326	−4.19	
	3.8006	−4.98	
	4.1407	3.52	

4. Safety testing of ST on MH-S cells

The cytotoxicity of ST on MH-S cells was evaluated using the CCK-8 assay. The cells were seeded in a 96 well plate with the density 1×10^4 cells/well. ST series solutions of 400, 200, 100, 50, 25, 12.5, and 6.25 μg/mL were prepared using RPMI 1640 medium. Each ST solution of 200 μL was added to individual wells of a 96-well plate and incubated for 12 h, followed by adding 15 μL of CCK-8 reagent to each well and further incubation for 1.5 h. The absorbance was measured at 450 nm by an enzyme-labeled instrument (Multiskan GO, Thermo Fisher Scientific, Waltham, MA, USA) to calculate the cell survival rate (n = 6). The same ST series solutions without cells as a control subtracted ST absorbance.

ST demonstrated moderate toxicity to MH-S cells, with cell viability above 90% at concentrations below 100 μg/mL (Figure S4).

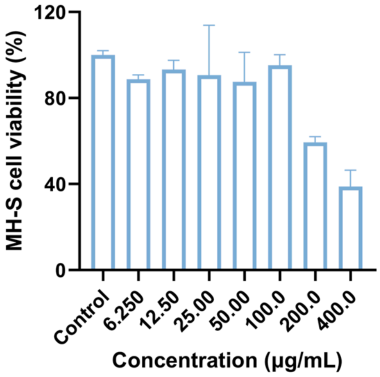

Figure S5. Cell viability of ST DPI against MH-S cells.

References

1. Fulmer, G.R.; Miller, A.J.M.; Sherden, N.H.; Gottlieb, H.E.; Nudelman, A.; Stoltz, B.M.; Bercaw, J.E.; Goldberg, K.I. NMR Chemical Shifts of Trace Impurities: Common Laboratory Solvents, Organics, and Gases in Deuterated Solvents Relevant to the Organometallic Chemist. *Organometallics* **2010**, *29*, 2176–2179, doi:10.1021/om100106e.
2. Gottlieb, H.E.; Kotlyar, V.; Nudelman, A. NMR Chemical Shifts of Common Laboratory Solvents as Trace Impurities. *The Journal of Organic Chemistry* **1997**, *62*, 7512–7515, doi:10.1021/jo971176v.

Disclaimer/Publisher's Note: The statements, opinions and data contained in all publications are solely those of the individual author(s) and contributor(s) and not of MDPI and/or the editor(s). MDPI and/or the editor(s) disclaim responsibility for any injury to people or property resulting from any ideas, methods, instructions or products referred to in the content.
